# Best Practices for Surgical Safety in Orthopaedics: A Comprehensive Review of Checklist Implementation

**DOI:** 10.7759/cureus.89476

**Published:** 2025-08-06

**Authors:** Ahmed Mohamed, Usman Fuad, Aasim Hagroo, Alaa Elasad

**Affiliations:** 1 Trauma and Orthopaedics, Royal Cornwall Hospital, Truro, GBR; 2 General Practice, Zagazig University, Zagazig, EGY

**Keywords:** never events, orthopaedic surgery, patient safety, surgical checklist, surgical safety, time-out procedure, who surgical safety checklist, wrong-site surgery

## Abstract

Despite advances in surgical techniques and technology, surgical complications in orthopaedics remain a significant patient safety concern, with preventable major errors continuing to occur. The implementation of surgical safety checklists and time-out procedures has emerged as a critical intervention to enhance patient safety and reduce complications.

This narrative review examines the role of surgical checklists and time-out procedures in reducing complications specific to orthopaedic surgery, analyzing their effectiveness, implementation challenges, and impact on patient outcomes. A comprehensive literature review was conducted examining peer-reviewed articles, systematic reviews, and clinical guidelines related to surgical safety checklists and time-out procedures in orthopaedic practice. Sources included studies on the available surgical safety checklist, never events data, and orthopaedic-specific safety interventions.

Evidence demonstrates that structured surgical checklists and time-out procedures significantly reduce wrong-site surgery, retained foreign objects, and communication-related errors in orthopaedic practice. Applying surgical safety checklists in practice has shown effectiveness in reducing overall surgical complications and mortality. However, compliance rates vary significantly across institutions and countries.

Surgical checklists and time-out procedures represent essential safety interventions in orthopaedic practice. While evidence supports their effectiveness in reducing complications and improving communication, successful implementation requires organizational commitment, staff education, and ongoing monitoring to ensure sustained compliance and optimal patient outcomes.

## Introduction and background

Orthopaedic surgery witnesses a great revolution, especially with the rapid advances in technology. Despite the significant advances in surgical techniques, implants, perioperative care, and efforts to decrease complications, complications continue to occur, which may result in serious, preventable adverse events known as never events. [1]. The complexity of orthopaedic procedures, combined with the need for precise anatomical identification and laterality confirmation, makes this specialty particularly vulnerable to certain types of surgical errors such as wrong-site surgery and retained surgical objects [2]. However, most of these complications are preventable through systematic safety approaches and measures.

The collaborative work of Gawande and colleagues led to the development of the World Health Organization (WHO) Surgical Safety Checklist in 2008 [3], marking a dramatic shift in perioperative safety culture and creating long-lasting safety guidelines that have been adopted globally [4,5]. This initiative was prompted by the evidence that surgical care, despite its benefits, carried substantial risks, with studies indicating that approximately half of all surgical complications were potentially preventable [6,7].

Orthopaedic surgery presents unique challenges that make using surgical safety checklists and time-out procedures particularly relevant [8]. The specialty often involves limb procedures, multiple levels in spinal surgery, and complex implant selections that need to have their validity checked for the operation, all of which increase the risk of human errors if not systematically verified at different stages of the patient journey until completion of the procedure. Analysis of never events data from the National Health Service (NHS) England between 2012 and 2020 revealed that orthopaedic surgery accounted for 460 of 3,247 reported never events, with wrong implants and wrong-site surgery being the most common categories [9,10].

This narrative review aims to examine the role of surgical checklists in reducing complications specific to orthopaedic surgery. We will discuss and analyze their effectiveness in preventing never events, evaluate implementation challenges and barriers in orthopaedic practice, assess their impact on patient outcomes, and identify strategies for optimization and future developments in surgical safety verification systems.

## Review

Methods

A comprehensive narrative review was conducted to examine the role of surgical checklists and time-out procedures in reducing orthopaedic complications. This review aimed to synthesize existing evidence on checklist effectiveness, implementation challenges, and clinical outcomes specific to orthopaedic surgery.

Search Strategy

Literature searches were performed using PubMed, MEDLINE, Cochrane Library, and Google Scholar databases from inception to August 2024. Search terms included combinations of the following: "surgical safety checklist", "WHO checklist", "time-out procedure", "orthopaedic surgery", "orthopaedic surgery", "never events", "wrong-site surgery", "patient safety", "surgical complications", "retained foreign objects", and "perioperative safety".

Inclusion Criteria

Peer-reviewed articles, systematic reviews, meta-analyses, clinical guidelines, and government reports related to surgical safety checklists in orthopaedic practice were included. Studies examining checklist effectiveness, implementation challenges, patient outcomes, and never events data were prioritized. Both prospective and retrospective studies were considered, along with qualitative research on implementation barriers and facilitators.

Exclusion Criteria

Non-English publications, case reports with fewer than 10 patients, studies focusing solely on non-orthopaedic specialties without relevant implications for orthopaedic practice, and opinion pieces without supporting evidence were excluded.

Study Selection

Articles were initially screened by title and abstract for relevance to surgical safety checklists and orthopaedic surgery. Full-text review was then conducted for potentially relevant studies. Additional sources were identified through reference lists of included studies, citation tracking, and expert recommendations from professional orthopaedic organizations.

Data Extraction

Key information extracted included study design, population characteristics, checklist implementation details, outcome measures (complications, mortality, compliance rates), implementation challenges, and clinical implications specifically relevant to orthopaedic practice. Particular attention was given to studies reporting never events data, wrong-site surgery incidents, and retained foreign object cases in orthopaedic settings.

Statistical Data Reporting

Statistical data from included studies are reported as originally published, including p-values, confidence intervals, and effect sizes where available. Statistical significance is clearly indicated, and non-significant findings are explicitly noted. When confidence intervals or p-values were not reported in the original studies, this is acknowledged in the text.

Quality Assessment

While formal quality assessment tools were not applied given the narrative nature of this review, preference was given to high-quality evidence including randomized controlled trials, large cohort studies, systematic reviews, and official government or professional organization reports. The strength of evidence and potential limitations were considered in the synthesis of findings.

Historical context and development

The recognition of medical errors as a major public health concern has been highlighted following the publication of "To Err is Human" by the Institute of Medicine in 1999, which estimated that medical errors caused between 44,000 and 98,000 deaths annually in American hospitals [11]. This report highlighted that healthcare workers are good people working in systems that need to be safer and catalyzed a global movement towards systematic approaches to patient safety, leading to the development of various safety interventions across healthcare disciplines.

The use of checklists and the development of structured approaches for safety-critical tasks have been formalized initially by aviation and then were imported to the healthcare system [12]. In surgical specialties, early efforts focused only on specialty-specific interventions, such as antibiotic prophylaxis protocols and venous thromboembolism prevention measures. However, the need for a comprehensive, universally applicable surgical safety framework became increasingly apparent as surgical volumes and their associated complications continued to grow globally.

The WHO's Safe Surgery Saves Lives campaign, launched in 2007 [3], was the first systematic attempt to develop globally applicable surgical safety standards. That campaign resulted in generating a 19-item checklist that was designed to be simple, universally applicable, and focused on checking the most common surgical morbidity and mortality factors across different healthcare systems. In orthopaedic surgery, several specialty-specific safety initiatives preceded the WHO checklist, including the American Academy of Orthopaedic Surgeons' "Sign Your Site" campaign [13] and the North American Spine Society's "Sign, Mark and X-ray" protocol [14], which demonstrated the historic safety challenges that orthopaedics faced and the successive clinical protocols trying to maintain safety.

Components of the WHO Surgical Safety Checklist: a three-phase approach

The WHO Surgical Safety Checklist is structured around three critical phases of surgical care: Sign In, Time Out, and Sign Out. Each phase addresses specific safety concerns directed towards different team members, creating multiple verification points throughout the perioperative process to maintain patient safety and support coordinated team performance during surgery [15]. Understanding the distinct purpose and implementation of each phase is essential for effective checklist utilization in orthopaedic surgery [16].

Sign In Phase: Pre-anaesthetic Verification

The Sign In phase occurs before the induction of anaesthesia and represents the initial safety verification point. This phase provides systematic confirmation of patient and procedural details. The primary objectives include verifying patient identity, confirming the intended surgical procedure and anatomical site, and ensuring the surgical site has been correctly marked.

The anaesthetic safety checks during Sign In include confirmation of known allergies, difficult airway assessment, aspiration risk evaluation, whether anticoagulant medications have been stopped or adjusted, whether the patient is diabetic, and whether a pregnancy test has been done in women. For orthopaedic patients, additional considerations may include assessment of positioning requirements, particularly for procedures requiring prone positioning or the use of tourniquets, the anticipated blood loss, and the potential risks associated with diathermy use. The verification of consent and availability of essential imaging studies, such as X-rays, MRI, or CT scans, ensures that all necessary information is available before proceeding with anaesthetic induction [17].

Time Out Phase: Immediate Pre-incision Verification

The Time Out represents the most critical verification point in the surgical safety checklist, occurring before skin incision when all team members are present and available. This phase requires full attention and active participation from all theatre team members [18]. Any safety concern must be highlighted in this stage [19].

This phase can be divided into four main categories. The first phase is the verification process; this phase focuses on team member introductions, establishing clear communication channels and defining roles for the procedure ahead, and the patient verification process that includes patient identity, surgical procedure, anatomical site, and positioning, whether a tourniquet is needed and what the pressure is.

The second phase is anticipated critical events, which include checks for the amount of blood loss anticipated, identifying any surgical or anaesthetic challenges, whether any investigations are required, and whether there is any backup plan if the primary plan fails.

The third phase is the anaesthetic check phase, which focuses mainly on patient concerns and comorbidities, American Society of Anesthesiologists (ASA) grade, whether monitoring equipment is required, whether the surgical site infection bundle has been undertaken, and whether venous thromboembolism (VTE) prophylaxis has been applied.

The last phase is the confirmatory phase, where we check that instruments are available and sterile, whether images are displayed, and whether any further X-rays are needed.

Sign Out Phase: Post-procedure Verification and Communication

The Sign Out phase occurs before the patient leaves the operating theatre and serves as the final verification point in the surgical safety process. This phase focuses on the communication of critical information that will impact postoperative care and ensures that all safety-critical tasks have been completed satisfactorily. The coordination of Sign Out typically involves the surgeon, anaesthetist, and nursing staff, with each contributing essential information about the completed procedure.

Procedure completion verification during Sign Out includes confirmation of the completed procedure name, review of any deviations from the planned procedure, and documentation of any complications or unexpected findings.

Instrument, swab, and sharp counting represents a critical safety verification during Sign Out, aimed at preventing retained foreign objects. The systematic counting of instruments, sponges, and other materials ensures that all items introduced during the procedure have been accounted for and removed. In orthopaedic surgery, where multiple instruments, drill bits, and guide wires may be used, this verification process becomes particularly important [20]. Moreover, if any specimens have been collected, they need to be correctly labelled at this stage [21]. Any problems with instruments or kits need to be highlighted as well in this stage to be addressed by the theatre managers.

Postoperative care planning communication during Sign Out ensures that all team members understand the immediate postoperative requirements and potential complications. This communication includes discussion of any postoperative instructions regarding post-procedural care, mobility restrictions, pain management plans, VTE prophylaxis needs, and monitoring requirements.

Current evidence on effectiveness

The landmark prospective study by Haynes et al. that included 7,688 patients across eight hospitals in different healthcare systems demonstrated the statistically significant effectiveness of the WHO Surgical Safety Checklist globally in reducing complications from 11% to 7% (p=0.003) and a statistically significant decrease in mortality from 1.5% to 0.8% (p=0.003) following checklist implementation [4]. While this study included all surgical specialties, it provides strong evidence for systematic checklist adoption across surgical disciplines, including orthopaedics.

Subsequent studies have specifically examined checklist effectiveness in orthopaedic populations. Sewell et al. conducted a prospective audit of 965 orthopaedic patients before and after implementation of an educational programme designed to increase WHO checklist use [21]. The study demonstrated a statistically significant improvement in checklist compliance from 7.9% to 96.9% (p<0.001) following education, though early complications and mortality rates showed statistically non-significant reductions from 8.5% to 7.6% (p=0.83) and 1.9% to 1.6% (p=0.88), respectively. The apparent discrepancy between improved compliance and modest clinical outcomes highlights important considerations about checklist effectiveness, particularly the distinction between completion rates and meaningful safety verification processes.

Implementation challenges

Hierarchy among the operative theatre staff might be a barrier to the proper implementation of a safe checklist. Recent research on psychological safety in operating rooms has shown that surgeons and anaesthetists may be reluctant to accept nurse leadership in checklist execution [22]. Active implementation of checklists requires every person in theatres to be supportive and well-educated about the importance of every step of a checklist, so team dynamics should be strengthened through structured, mandatory training programmes.

Some team members might find it inconvenient, thinking that surgical checklists cause delays and interrupt the workflow, so they might show reluctance or rush when performing them. Studies examining workflow impact have identified this as a significant barrier to effective implementation [23]. The clinical importance of these delays and time consumption must be highlighted to avoid serious, preventable adverse events. Additionally, literature has proved that using checklists doesn't have a great impact on theatre time [24,25].

Increased workload and preoccupation with concurrent activities is another considered challenge, especially during the Time Out process when every theatre attendee will be busy multitasking. Surgeons prepare themselves for surgery by positioning the patients, readjusting theatre lights, displaying radiographs, and getting scrubbed. Scrub nurses prepare themselves for the operation by checking their kits, prepping, and draping tools. Anaesthetists adjust their monitors and prepare the patient for positioning and surgery. Checklist compliance sometimes interrupts this work routine and adds to the existing workload. Similarly, Sign Out is another significant barrier where surgeons are rushing to complete the postoperative notes and to rest before starting the next case, nurses are busy packing and counting their instruments, and the anaesthetist is busy awakening the patient; as a result, key postoperative concerns or plans might not be discussed.

Emergency cases represent a significant challenge as team members will be rushing to save lives. However, the rate of human errors is higher in these settings. Application of surgical checklists in emergent and urgent situations has proven to statistically significantly decrease complications by 36% (95% CI: 24-47%; p<0.001) and decrease mortality by 62% (95% CI: 44-74%; p<0.001) [26].

Finally, patients might find it inappropriate and anxiety-provoking to listen to a full 19-item checklist immediately before a high-stress event such as surgery, especially if anyone raises a concern or when talking about blood loss. A systematic review of checklist implementation found that patient acceptance and understanding of safety procedures vary significantly across different healthcare settings [27]. Therefore, educating patients about the checklist and explaining that team members will be doing some checks for the smooth running of the operation and to prevent any problems that might happen usually makes them more relaxed.

Impact on specific complications

Never events represent the worst scenarios of preventable surgical complications in orthopaedic practice. Analysis of NHS England's never events data provides valuable insights into the types and frequencies of preventable complications. Wrong implant incidents accounted for 206 of 460 orthopaedic never events between 2012 and 2020 [9], with wrong hip and knee prostheses representing most of these incidents. These events highlight that orthopaedic surgery is highly vulnerable to implant-related errors and the importance of adhering to systematic checklists throughout the perioperative process in orthopaedic procedures.

Wrong-site surgery represents one of the most devastating preventable complications in orthopaedic practice. Orthopaedic procedures represent approximately one-third of wrong-site surgery incidents in the NHS England dataset [9]. Reviewing the National Patient Safety Agency database for wrong-site surgery incidents in orthopaedics revealed 133 orthopaedic wrong-site incidents [28,29]. Researchers estimated that 83% of actual harm cases and 15% of near-miss events could have been prevented through proper checklist use (95% CI: 76-89% for harm cases, 95% CI: 9-23% for near-miss events) [30]. This finding underscores the particular importance of systematic verification procedures in orthopaedic practice. Effective laterality verification requires multiple confirmation points throughout the perioperative process, including preoperative site marking and confirming with the patient which side the procedure is intended for before starting, such as in cases of joint injection or aspiration.

Retained surgical objects represent another category of never events with particular relevance to orthopaedic surgery. The complex instrumentation used in many orthopaedic procedures, including drill bits, wire guides, and temporary implants, creates multiple opportunities for retention if not systematically tracked. Effective prevention requires systematic counting procedures, clear communication protocols, and enhanced verification during the sign-out phase of surgical checklists [31].

Strategies for optimization

Successful implementation of surgical checklists and time-out procedures requires committed leadership at multiple organizational levels. Championing is a key factor in the successful implementation of safety checklists [32,33]; champions should be selected carefully and should build a team who can effectively implement the strategy across hospitals. Surgeons play a particularly crucial role in modelling desired behaviours and encouraging team participation through regular communication about safety goals and adequate resource allocation.

Comprehensive education programmes are also very important in addressing the rationale behind using checklists, explaining their purpose and demonstrated outcomes. Effective programmes typically include didactic education about checklist effectiveness evidence, including the WHO Surgical Safety Checklist in mandatory training, simulation training for practical skill development, and multidisciplinary approaches involving all team members. The dramatic improvement in compliance demonstrated by Sewell et al. [21] following targeted education illustrates the potential impact of well-designed training initiatives.

Audits should be conducted regularly to monitor team compliance with checklist adherence before orthopaedic procedures; these audits should be presented, and solutions for any problems need to be resolved to maintain the quality of care.

Future directions

Emerging technologies offer potential enhancements to traditional checklists and time-out procedures. Artificial intelligence-powered systems can analyze patient information and identify potential discrepancies, while radio-frequency identification tracking of implants and instruments can provide real-time verification. Evidence suggests that the application of artificial intelligence technology improved the efficiency of checklist execution and decreased both never events and healthcare workers' workload [34]. Computer vision systems may supplement human verification processes, and mobile applications can provide portable checklist platforms with enhanced functionality.

Research is exploring extensions of traditional checklist concepts, including "second time-out" protocols for procedures lasting more than 3-4 hours, systematic verification protocols for complex multi-stage procedures, and adaptation of checklist principles to outpatient and office-based settings. Future development also involves integration of checklist data with broader quality improvement initiatives, including risk stratification, predictive analytics, and comparative effectiveness research.

## Conclusions

Surgical checklists and time-out procedures represent essential components of comprehensive safety programmes in orthopaedic surgery. The evidence clearly demonstrates their effectiveness in reducing wrong-site surgery, retained foreign objects, and communication-related complications. However, successful implementation requires committed leadership, comprehensive education, systematic monitoring, and continuous improvement efforts. The quality of checklist implementation is more important than simple completion rates, requiring meaningful team engagement and thorough verification processes.

Orthopaedic surgery's unique characteristics mandate using systematic safety verifications. Future developments will likely involve enhanced verification technologies and integration with broader quality improvement initiatives, but the fundamental principle of systematic safety verification through structured team communication will remain central to preventing complications and improving patient outcomes.
